# Addressing mental health gaps through service innovation, workforce development and policy

**DOI:** 10.1192/bji.2026.10113

**Published:** 2026-08

**Authors:** Marinos Kyriakopoulos

**Affiliations:** 1 Department of Psychiatry, National and Kapodistrian University of Athens, Athens, Greece.; 2 Department of Child and Adolescent Psychiatry, Institute of Psychiatry, Psychology and Neuroscience, King’s College London, London, UK

Mental health systems across the world face substantial challenges in meeting growing and increasingly complex population needs. At the same time, many health systems are exploring innovative and scalable approaches to improve access to care, strengthen frontline services and address broader determinants of mental health. Several papers in this issue of *BJPsych International* collectively reflect these intersecting priorities. Together, they examine unmet mental health needs in underserved populations, present novel models of service delivery, consider ways to bridge training gaps and discuss the role of policy as part of a wider public mental health strategy.

In this regard, Aboaja and colleagues^1^ present the mental health landscape associated with limited resources and workforce shortages in Guatemala, discuss opportunities to promote effective access to mental health services and make suggestions aiming to improve resource allocation. Peritogiannis and colleagues^2^ highlight the importance of mobile mental health units, locally based generic community mental health teams delivering evidence-based interventions, in rural Greece; examine their relevance for mental healthcare and policy in remote and underserved areas; and discuss the challenges and opportunities of this community-based initiative. Al-Mahrouqi and colleagues^3^ present on the expansion of telehealth and telepsychiatry in Oman, its regulatory framework and associated policy developments, and offer valuable insights on how technology may assist in wider reach and more cost-effective services. Focusing on the upskilling of the workforce, Humayun and Najmussaqib^4^ report on the process and evaluation of training primary healthcare workers in Khyber Pakhtunkhwa in Pakistan. This involved a 5-day training session for which the Mental Health Gap Action Programme Humanitarian Intervention Guide was contextualised into a guide adapted for use in Pakistan, and developed into a mobile application. The results of their investigation demonstrated significant improvements in participants’ knowledge, sustained over time, which argues in favour of such capacity-building initiatives.

Although differing in context and methodology, the above articles, among many others in this issue, share a common concern: how mental health systems can better identify need, extend access to care, strengthen frontline capacity and address broader determinants of mental health across diverse contexts. I hope you enjoy it!
